# Application of the mild behavioral impairment checklist in Chinese patients with the behavioral variant of frontotemporal dementia

**DOI:** 10.1007/s10072-023-07049-4

**Published:** 2023-09-05

**Authors:** Yue Cui, Li Liu, Min Chu, Kexin Xie, Zhongyun Chen, Haitian Nan, Yu Kong, Tianxinyu Xia, Yingtao Wang, Yihao Wang, Qianqian He, Liyong Wu

**Affiliations:** Department of Neurology, Xuanwu Hospital, Capital Medical University, Changchun Street 45, Beijing, 100053 China

**Keywords:** Behavioral variant of frontotemporal dementia, Mild behavioral impairment checklist, Neuropsychiatric inventory questionnaire, Frontal behavioral inventory, Neuropsychiatric symptoms

## Abstract

**Background:**

The mild behavioral impairment checklist (MBI-C) designed to capture neuropsychiatric symptoms in the whole spectrum of elder with or without dementia, have been verified in mild behavioral impairment, mild cognitive impairment and Alzheimer's Disease, but never used in the behavioral variant of frontotemporal dementia (bvFTD).

**Methods:**

Fifty-two patients with bvFTD (mild, *n* = 30; moderate-severe, *n* = 22) and 82 community-dwelling elderly individuals (HCs) were enrolled. All subjects were assessed with a full neuropsychological scale including the MBI-C, Neuropsychiatric Inventory Questionnaire (NPI-Q), and Frontal Behavioral Inventory (FBI). Receiver operating characteristic curves were drawn to analyze the sensitivity and specificity of the MBI-C, NPI-Q, and FBI, and cutoff points were determined using the Youden index.

**Results:**

The MBI-C and domain scores in all patients with bvFTD were significantly higher than those in HCs. The most common symptoms of bvFTD were *apathy* (82.7%) and *impulse dyscontrol* (80.8%). The MBI-C score was positively correlated with the NPI-Q, FBI, and Activities of Daily Living. For differentiating patients with both bvFTD and mild bvFTD from HCs, the optimal MBI-C cutoff point was 5.5 with a sensitivity of 100% and specificity of 82%, and its sensitivity was higher than that of the NPI-Q and FBI.

**Conclusion:**

The MBI-C is a sensitive tool for screening behavioral and psychological symptoms in patients with bvFTD, even in the early stages of the disease.

**Supplementary Information:**

The online version contains supplementary material available at 10.1007/s10072-023-07049-4.

## Introduction

Behavioral variant frontotemporal dementia (bvFTD) is the commonest subtype of frontotemporal lobar degeneration, characterized by personality and behavioral changes in the early stages that gradually worsen over the course of the disease and are accompanied by cognitive impairment [1]. At present, the most commonly used scales assessing behavioral and psychological symptoms for early identification and course monitoring of bvFTD are the Neuropsychiatric Inventory Questionnaire (NPI-Q) and the Frontal Behavioral Inventory (FBI) [2, 3]. However, with the discovery of mild behavioral impairment as a pre-onset stage of dementia, a psychobehavioral symptom assessment tool that focuses on mild behavioral impairment may be more sensitive for the early screening of bvFTD [4].

The Mild Behavioral Impairment Checklist (MBI-C) was developed by Professor Zahinoor Ismail et al. [5] in Canada in 2017 by the modified Delphi method to evaluate neuropsychiatric symptoms for early screening of mild behavioral impairment. Currently, the MBI-C has been translated into several languages and is widely used for screening of mild behavioral impairment, subjective cognitive decline, mild cognitive impairment, and Alzheimer's disease (AD), where it has been confirmed to have good reliability and validity [6–12]. However, as a sensitive psychobehavioral symptom assessment tool, the MBI-C has never been reported to be used in research pertaining to the bvFTD population. In previous literature, only one patient with mild behavioral impairment who eventually evolved into bvFTD had received the MBI-C to describe his symptoms [13]. In that reported case, the MBI-C seemed to perform better than NPI-Q in detecting impulse dyscontrol [13]. Combined with our past research, which has confirmed that the sensitivity and specificity of the MBI-C is higher than the NPI-Q in distinguishing AD from the healthy elderly population, we believed that the MBI-C would be a more effective tool for screening behavioral and psychological symptoms in patients with bvFTD [12].

In this study, patients with bvFTD and the community-dwelling elderly population were enrolled to explore whether the MBI-C can sensitively reflect the characteristics of behavioral and psychological symptoms in patients with bvFTD and to verify whether the MBI-C is suitable for screening Chinese patients with bvFTD, especially in its early stages.

## Methods

### Participants

Fifty-two patients with bvFTD, whose ages ranged from 50 to 80 years, were recruited from the memory clinic at Xuanwu Hospital. All patients met the 2011 International Behavioural Variant FTD Criteria Consortium (FTDC) diagnostic criteria [14] for probable bvFTD and ruled out other forms of dementia. According to the score of Clinical Dementia Rating (CDR) [15], they were classified into 30 patients with mild bvFTD (CDR = 1) and 22 patients with moderate-severe bvFTD (CDR = 2 or 3). In addition, 82 community-dwelling elderly individuals aged ≥ 50 years without subjective cognitive complaints were recruited as healthy controls (HCs) from several communities in Beijing. Elderly community individuals were excluded if they had a medical condition that could cause cognitive and behavioral symptoms, had a previous history of alcohol or drug abuse, or were otherwise unable to cooperate with general medical examination. All participants underwent clinical and neuropsychological examinations by experienced neurologists within two weeks of enrollment. Written informed consent from the individuals themselves or their families for participation in this study was obtained according to the Declaration of Helsinki. This study was approved by the Ethics Committee of Xuanwu Hospital, Capital Medical University, Beijing, China.

### Neuropsychiatric and cognitive evaluations

Demographic information was obtained from participants or their caregivers. The Chinese versions of the Mini Mental State Examination (MMSE) [16] and the Montreal Cognitive Assessment (MoCA) [17] were used to evaluate general cognitive function. Specific cognitive domains such as memory, language, attention, and executive function were tested using the Chinese version of the Auditory Verbal Learning Test (AVLT) [18], Boston Naming Test (BNT) [19], Trail Making Test A (TMT-A) [20], and Trail Making Test B (TMT-B) [20] separately. In addition, Activities of Daily Living (ADL) [21] was used to assess the ability of living and CDR [15] was used to evaluate the severity of dementia. We used the Chinese version of the MBI-C [12], NPI-Q [22], and FBI [2] to assess neuropsychiatric symptoms.

The MBI-C includes 34 items and is divided into five domains: 1) interest, motivation, and drive; 2) mood or anxiety symptoms; 3) the ability to delay gratification and control behavior, impulses, oral intake, and/or changes in reward; 4) following societal norms and having social graces, tact, and empathy; and 5) strongly held beliefs and sensory experiences [5]. According to previous literature, these five domains were abbreviated as *Apathy*, *Impulse Dyscontrol*, *Mood/Anxiety*, *Social Inappropriateness,* and *Psychosis* [23]. This scale, which can be completed by the patient, caregiver, or clinician, assesses whether the subject has persistent or intermittent neuropsychiatric symptoms for more than 6 months and evaluates the severity of the symptoms on a 3-point scale (1 = mild, present but not obvious; 2 = moderate, obvious but not very prominent; 3 = severe, very prominent) [5]. The total score ranges from 0 to 102 [5]. With the consent of the original author, MBI-C was translated into Chinese with high reliability and validity by our team in 2019 [12].

### Statistical analyses

SPSS version 26.0 was used for data analysis, and GraphPad Prism 7 was used for the creation of graphics. Owing to the unknown distribution type of the population and the small sample size of the study group, the Kolmogorov–Smirnov test was used to verify whether the data were normally distributed, and the results showed that only age in each group followed a normal distribution. Descriptive statistics for each group were reported using the χ2 test (sex and prevalence rate), independent samples *t*-test when assumptions of normality, variance, and independence were met (age), and Wilcoxon-Mann–Whitney U test for non-normal continuous variables (other demographic characteristics and neuropsychological scales). Spearman's rank correlation coefficient was calculated to evaluate the correlation between age, years of education, disease duration, scores on other neuropsychological scales, and MBI-C scores. Receiver operating characteristic (ROC) curves were drawn to analyze the sensitivity and specificity of the MBI-C, the area under the curve (AUC) was calculated, and its cutoff points were determined using the Youden index, as compared with the NPI-Q and the FBI. The level of statistical significance was set at *p* = 0.05.

## Results

### Demographic and general clinical information of the sample

All 134 participants offered complete demographic information and completed the full set of neuropsychological scales, including the assessment of neuropsychiatric symptoms, cognitive function, and daily living ability. The descriptive parameters of all bvFTD, mild bvFTD, moderate-severe bvFTD, and HC groups are summarized in Table 1. There were significant differences between the HC and all bvFTD groups in terms of sex (χ^2^ = 4.21, *p* = 0.040) and years of education (U = 1528.5, *p* = 0.018), whereas there was no between-group difference in age (t = 1.02, *p* = 0.311). The disease duration in the bvFTD group was 2 (1–3) years, while that of the mild bvFTD group and moderate-severe bvFTD group were 2 (1–4) years and 2 (1–3) years, respectively. The HC group performed better than all the bvFTD groups on the results of each scale and MBI-C domain. Most of the scale scores showed significant differences between the mild bvFTD group and the moderate-severe bvFTD group except for the BNT score, MBI-C total and partial domain scores (*mood and anxiety*, *impulse dyscontrol*, and *social inappropriateness*), and NPI-Q score. The neuropsychological scale results are shown in Table 1.

**Table 1:** Demographic characteristics and psychometric results

	HC (*n* = 82)	All bvFTD (*n* = 52)	p_1_	bvFTD		p_2_
				mild (*n* = 30)	moderate-severe (*n* = 22)	
Sex (male/female)	25/57	25/27	0.040^*^	13/17	12/10	0.424
Age (years)	62.9 ± 9.1	61.2 ± 9.5	0.311	60.2 ± 9.7	62.6 ± 9.2	0.373
Years of education	12 (9–15)	9 (7–14.3)	0.018^*^	9 (8.5–14.5)	12 (5.5–14.5)	0.929
MMSE	29 (28–30)	16.5(13–22)	< 0.001^*^	21 (15.3–25.8)	13 (8.3–15.8)	< 0.001^*^
MoCA	26 (24–27)	10 (5.5–16)	< 0.001^*^	15 (10–16.5)	5 (3.3–9.5)	< 0.001^*^
TMT A time (s)	50 (38–63)	108(72–150)	< 0.001^*^	100 (65.5–128.5)	150 (90–150)	0.014^*^
TMT B time (s)	76 (57.3–100)	200 (90–300)	< 0.001^*^	119 (89.5–300)	300 (291.8–300)	0.022^*^
AVLT learning (45)	24 (19–28)	11 (4.5–14.5)	< 0.001^*^	13 (8–16.8)	5 (2.5–9.5)	0.001^*^
AVLT delayed recall (15)	8 (6–11)	0 (0–2.3)	< 0.001^*^	0 (0–5)	0 (0–0)	0.017^*^
AVLT cued recall (15)	11 (10–13.8)	1 (0–4)	< 0.001^*^	1.5 (0–5)	0 (0–1)	0.006^*^
BNT (30)	25 (22–27)	10 (5.3–16.8)	< 0.001^*^	10 (5.5–18.5)	8 (5–16)	0.404
ADL	20 (20–20)	31 (22.3–43.8)	< 0.001^*^	23.5 (21–30.3)	47.5 (39–58.3)	< 0.001^*^
Sum of CDR	0 (0–0)	9.3(5.6–13.4)	< 0.001^*^	6 (4.4–7.5)	13.8 (12.4–17)	< 0.001^*^
MBI-C total score	0 (0–3)	20 (15–31)	< 0.001^*^	20 (13–29.5)	20 (17.3–35.5)	0.258
MBI-C apathy	0 (0–0)	7.5(2–10.8)	< 0.001^*^	4 (0.8–8)	10 (8.3–13)	< 0.001^*^
MBI-C mood and anxiety	0 (0–0)	2(0–4)	< 0.001^*^	3 (0–5)	2 (0–4)	0.302
MBI-C impulse dyscontrol	0 (0–2)	5 (6–11)	< 0.001^*^	5.5 (2.8–11.3)	3 (0–11.3)	0.293
MBI-C social inappropriateness	0 (0–0)	1(0–3)	< 0.001^*^	1 (0–3)	1.5 (0–3.3)	0.708
MBI-C psychosis	0 (0–0)	0 (0–1)	0.001^*^	0 (0–0)	0.5 (0–2.3)	0.019^*^
NPI-Q	0 (0–2)	7(5–11)	< 0.001^*^	6.5 (4–9)	8.5 (5–14.8)	0.163
FBI	0 (0–1)	23(13–30.8)	< 0.001^*^	19 (11.8–24.5)	28 (23.8–40.3)	< 0.001^*^

Descriptive parameters are shown as mean ± SD or median (quartile); p_1_: Statistical analysis was performed between all bvFTD and HC groups; p_2_: Statistical analysis was performed between the mild and moderate-severe bvFTD groups; * Statistical differences were found

*ADL* Activities of daily living; *AVLT* Auditory verbal learning test; *BNT* Boston naming test; *bvFTD* Behavioral variant frontotemporal dementia; *CDR* Clinical dementia rating; *FBI* The frontal behavioral inventory; *HC* Healthy control; *MBI-C* Mild behavioral impairment checklist; *MMSE* Mini mental state examination; *MoCA* Montreal cognitive assessment; *NPI-Q* Neuropsychiatric inventory questionnaire; *TMT* Trail making test

### Total and domain frequencies of the Mild Behavioral Impairment-Checklist

**Fig. 1:**
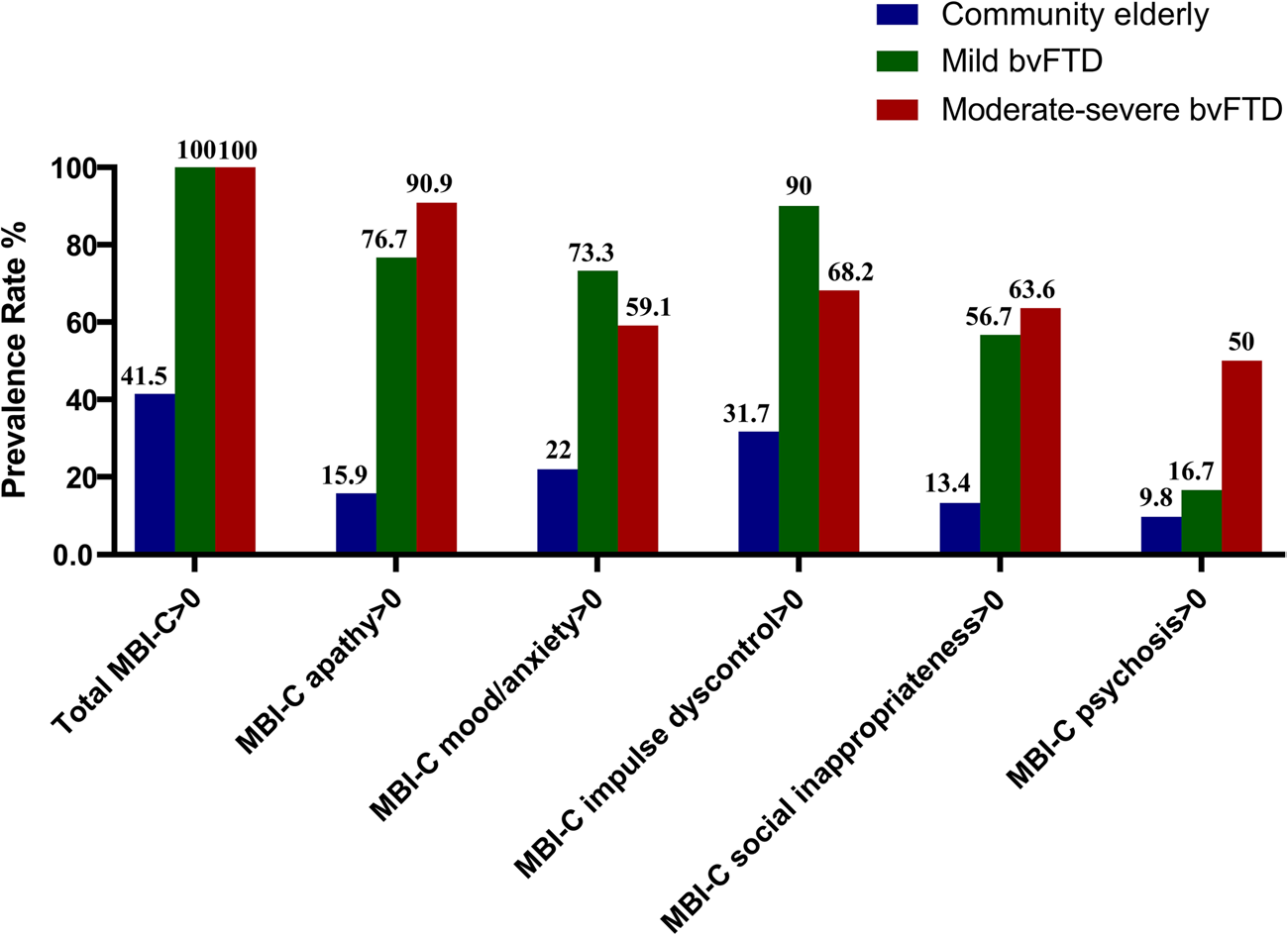
The prevalence rate of MBI-C symptoms

In this study, all patients with bvFTD and 41.5% of HCs had MBI-C scores greater than 0. The prevalence rate of each MBI-C symptoms in all bvFTD from high to low was *apathy* (82.7%), *impulse dyscontrol* (80.8%), *mood/anxiety* (67.3%), *social inappropriateness* (59.6%), and *psychosis* (30.8%). When patients with bvFTD were divided by disease severity, we found the highest incidence of *impulse dyscontrol* (90%) in mild bvFTD and *apathy* (90.9%) in moderate-to-severe bvFTD. In the MBI-C domains, except for *psychosis*, the prevalence rates of mild and moderate-severe bvFTD were significantly higher than those in the HC group, while no difference was found between the mild and moderate-severe bvFTD groups. There was no significant difference in the prevalence rate of *psychosis* between the HC group and the mild bvFTD group, but it was significantly higher in the moderate-severe bvFTD group (χ^2^ = 19.15, *p* < 0.001). The prevalence rates of each MBI-C symptom in the HC, mild bvFTD, and moderate-severe bvFTD groups are presented in Fig. 1.

### Correlation between the Mild Behavioral Impairment-Checklist and other scales

**Fig. 2:**
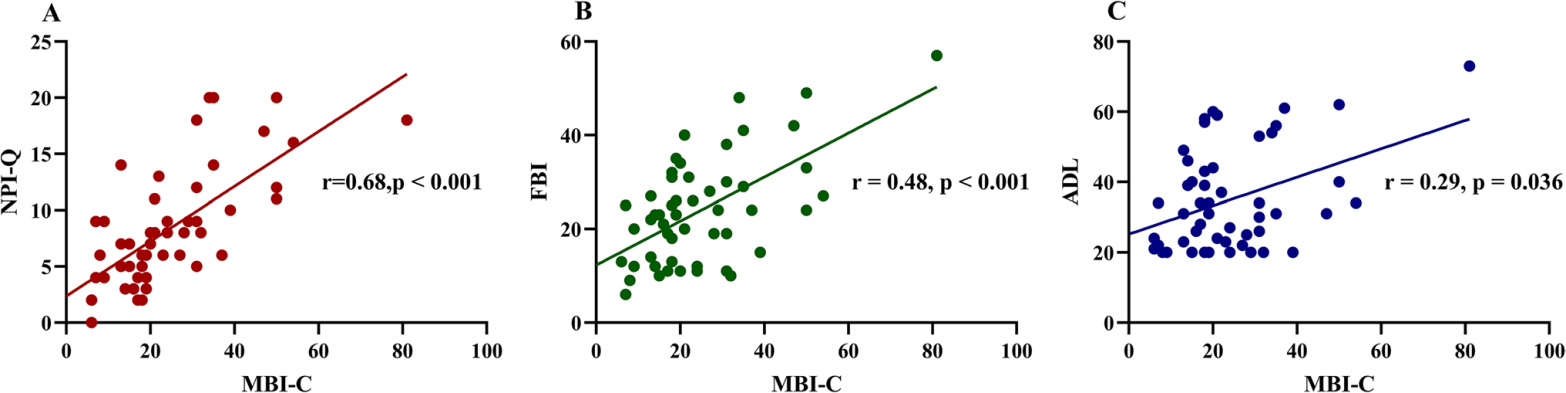
Correlation between MBI-C and NPI-Q (**A**), FBI (**B**), and ADL (**C**)

All correlation analyses were performed for the bvFTD group. There was no significant correlation between the total MBI-C score and age, sex, years of education, or disease duration, as determined by Spearman’s correlation test (r = -0.2, *p* = 0.152; r = -0.14, *p* = 0.113; r = 0.12, *p* = 0.418; and r = -0.02, *p* = 0.898, respectively). The MBI-C score was positively correlated with the total score on the NPI-Q (r = 0.68, *p* < 0.001), FBI (r = 0.48, *p* < 0.001), and ADL (r = 0.29, *p* = 0.036), as shown in Fig. 2. There was no correlation between the MBI-C and MMSE scores (r = 0.06, *p* = 0.691), MoCA (r = 0.02, *p* = 0.882), AVLT learning (r = -0.07, *p* = 0.651), AVLT Delayed recall (r = -0.04, *p* = 0.815), AVLT Cued recall (r = 0.05, *p* = 0.769), TMT-A (r < -0.01, *p* = 0.982), TMT-B (r = 0.08, *p* = 0.679), BNT (r < -0.01, *p* = 0.995), or the sum of CDR (r = 0.24, *p* = 0.089).

### The Mild Behavioral Impairment-Checklist for detecting bvFTD

**Fig. 3:**
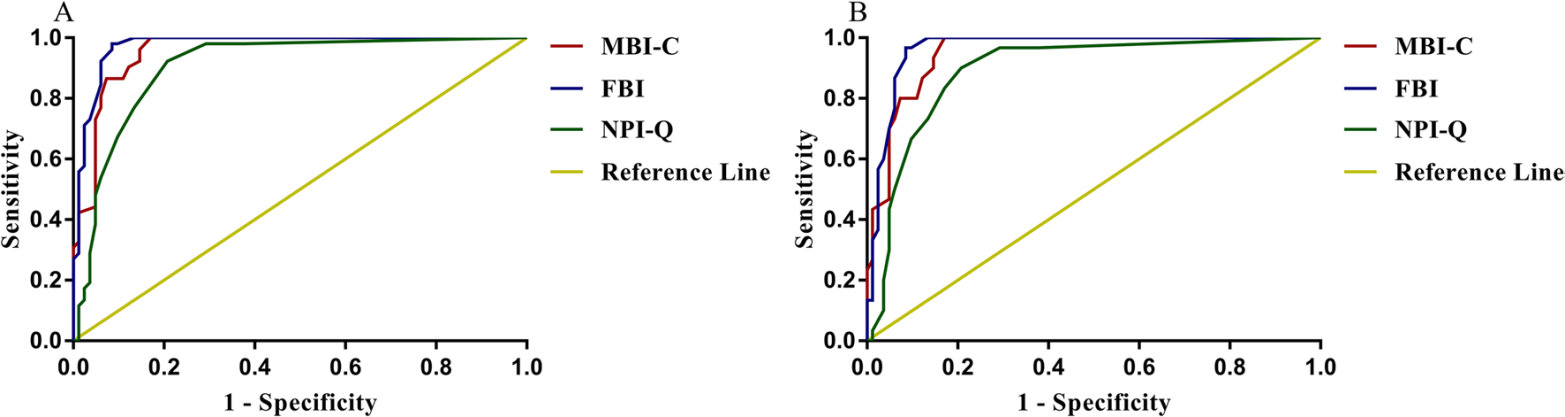
Receiver operating characteristic curves using the MBI-C, NPI-Q, and FBI. **A** differentiating all bvFTD from the community elderly; **B** differentiating mild bvFTD from the community elderly

For distinguishing all bvFTD from the HC group, ROC analysis revealed that the AUC and 95% confidence intervals (95% CI) of the MBI-C, NPI-Q, and FBI was 0.96 (0.93–0.99), 0.91 (0.85–0.96), and 0.98 (0.95–1), respectively. The AUC of the MBI-C and FBI was not statistically significant, whereas that of the NPI-Q was significantly lower. The optimal cutoff point of the MBI-C was 5.5, with a sensitivity of 100% and specificity of 83%. When the optimal cutoff point of the FBI was 8, its specificity was higher than that of the MBI-C, but its sensitivity was lower. In addition, the optimal cutoff point of the NPI-Q was 2.5, which yielded both lower sensitivity and specificity for discriminating bvFTD from the HC group than those of MBI-C and FBI. For distinguishing mild bvFTD from the HC group, the AUC of the MBI-C (0.95) and FBI (0.97) was not statistically significant, while that of the NPI-Q (0.89) was significantly lower. The optimal cutoff points and screening specificity were consistent with the situation in distinguishing bvFTD from the HC group, while the sensitivity of the NPI-Q and FBI further decreased to 90% and 97%, respectively. The results are shown in Fig. 3 and Table 2.

**Table 2:** Optimal cutoff points and validity of the MBI-C, NPI-Q, and FBI

	bvFTD and community elderly				Mild bvFTD and community elderly			
	AUC	Cutoff points	Sensitivity	Specificity	AUC	Cutoff points	Sensitivity	Specificity
MBI-C	0.96	5.5	100%	83%	0.95	5.5	100%	83%
NPI-Q	0.91	2.5	93%	79%	0.89	2.5	90%	79%
FBI	0.98	8	98%	91%	0.97	8	97%	91%

*AUC* Area under the curve; *bvFTD* Behavioral variant frontotemporal dementia; *FBI* Frontal behavioral inventory; *MBI* Mild behavioral impairment; *MBI-C* Mild behavioral impairment checklist; *NPI-Q* Neuropsychiatric inventory questionnaire

The optimal cutoff point in the MBI-C was up to 12 when distinguishing the moderate-severe bvFTD group from the HC group, with decreased sensitivity and increased specificity. The results are presented in Additional file 1 and Additional file 2. When distinguishing mild bvFTD from moderate-severe bvFTD group, both AUCs of the MBI-C and NPI-Q were below 0.7 (0.59 and 0.61, respectively), while the AUC of the FBI was 0.79.

## Discussion

To the best of our knowledge, this is the first study to explore the application of the MBI-C in patients with bvFTD. We found that the MBI-C can screen for bvFTD in its early stage in healthy elderly individuals, and its optimal cutoff point was 5.5, with excellent sensitivity and good specificity.

The MBI-C was specifically developed to assess mild behavioral impairment, a predementia stage initially proposed in the context of early frontotemporal dementia [24]. This makes the MBI-C a promising clinical tool for sensitive identification of the psychobehavioral symptoms in patients with bvFTD. In contrast to the NPI-Q and FBI, the domain of the MBI-C is designed completely according to the mild behavioral impairment structure [5]. *Impulse dyscontrol* mainly includes the question about disinhibition and perseverative, and *social inappropriateness* mainly includes the question about disinhibition and loss of empathy [5]. In our study, as determined by the MBI-C, the most prevalent domain encountered in mild bvFTD was *apathy* and in moderate-severe bvFTD was *impulse dyscontrol.* Both *mood/anxiety* and *social inappropriateness* were present in more than half of the patients with mild and moderate-severe bvFTD, respectively. This performance in the MBI-C is completely consistent with the frequency of indicators for possible bvFTD in FTDC criteria [14], which identified apathy as the most common symptom of bvFTD, followed by disinhibition, perseverative, and loss of empathy. Furthermore, compared with dementia mostly composed of AD reported in the previous literature, in both mild and moderate-severe bvFTD, the prevalence of *apathy* (76.7% and 90.9% versus 68.38%), *impulse dyscontrol* (90.0% and 68.2% versus 64.96%), and *social inappropriateness* (56.7% and 63.6% versus 38.03%) were higher [23]. This suggests that MBI-C can effectively reflect the symptom characteristics of patients with bvFTD.

In this study, the sex ratio and years of education between the bvFTD and HC groups were not exactly matched, but previous studies have shown that this did not affect NPS scores of the neuropsychiatric scale [25]. Our study also suggested that the MBI-C score of bvFTD is a relatively independent indicator, and there is no significant correlation between MBI-C score and age, sex, years of education, course of disease, or any cognitive function. This may indicate that the MBI-C score can specifically reflect the psychobehavioral symptoms of patients with bvFTD without the influence of other unrelated variables. Furthermore, the MBI-C score is positively correlated with the NPI-Q and FBI scores, but the correlation coefficient with NPI-Q is larger, which is probably because the FBI contains some items that are correlated with cognitive ability (such as decreased attention and decreased ability of organization) rather than psychobehavioral symptoms [26]. In addition, the MBI-C score was found to have a weak correlation with ADL score, which may be due to the fact that the daily living ability of patients with bvFTD is affected by psychobehavioral symptoms to a certain extent.

In previous studies, the MBI-C has been widely used in the screening of mild behavioral impairment and AD, and has been proven to have good screening ability [6, 12]. Several studies have screened mild behavioral impairment in populations with subjective cognitive decline or mild cognitive impairment, with the optimal cutoff point ranging from 6.5 to 8.5, the common feature being that the sensitivity of screening is higher than the specificity [6, 7]. In addition, as early as 2019, our research team translated the original MBI-C into Chinese, and found that the optimal cutoff point for identifying AD dementia was 6.5, with a sensitivity of 86.96% and specificity of 86.00% [12]. Screening ability of bvFTD is an unexplored area for the MBI-C.

In this study, we further found that the optimal cutoff point of the MBI-C for screening all patients with bvFTD was 5.5, with a sensitivity of 100% and specificity of 83%. Furthermore, when screening patients with mild bvFTD from the HC group, the optimal cutoff point, sensitivity, and specificity of the MBI-C did not change compared with screening all patients with bvFTD. This is probably related to our limited sample size, but it also suggests that the MBI-C is likely to have a good early screening ability for bvFTD.

Our study also found that MBI-C may be more suitable for bvFTD screening than NPI-Q, but whether MBI-C has higher sensitivity than FBI is still questionable. Both the NPI-Q and FBI are commonly used for early screening and disease course monitoring of bvFTD, and they have good reliability and validity with high detection ability [3, 27, 28]. By calculating the AUC, we found that the detection ability of the MBI-C for bvFTD (mild, moderate-severe, or both) was consistent with that of FBI, and higher than that of the NPI-Q. The NPI-Q has lower sensitivity and specificity than the MBI-C and FBI in screening bvFTD, which is likely due to its overcomprehensive coverage of psychobehavioral symptoms and insufficient specificity for bvFTD [22, 29]. When the optimal cutoff point was taken for comparison, the MBI-C showed higher sensitivity and lower specificity in the screening of bvFTD(or mild bvFTD) than the FBI, suggesting that the MBI-C may be more suitable for screening of bvFTD, while the FBI is more suitable for confirming the clinical diagnosis of bvFTD. The unchanged sensitivity in the MBI-C and decreased sensitivity in the NPI-Q and FBI when distinguishing mild bvFTD rather than all bvFTD from HC also corroborates this point. However, the ROC curve showed that the sensitivity and specificity of FBI in screening bvFTD were higher than those of MBI-C in most cases if the optimal cut-off point was not considered. The MBI-C is a psychobehavioral scale specifically designed for the predementia stage, and further expansion of the sample size is warranted to clarify which of the MBI-C or the FBI has higher sensitivity in screening for bvFTD [30].

The main limitation of this study is the small sample size, and it is necessary to further expand the number of subjects to reduce sampling error. In addition, it should be noted that most patients with bvFTD in this study voluntarily sought medical treatment due to obvious symptoms, which may affect the accuracy of our results. Further long-term evaluation of patients with bvFTD is necessary to confirm the ability of the MBI-C to monitor the course of the disease.

Our study showed that, compared with the NPI-Q, the Chinese version of the MBI-C was more sensitive and specifitive for screening patients with bvFTD, especially in its early stage. The MBI-C is expected to be a tool for bvFTD screening with high sensitivity and specificity as FBI.

### Supplementary Information

Below is the link to the electronic supplementary material.

Supplementary file1 (DOCX 111 KB)

## Data Availability

The data supporting the findings of this study are available on request from the corresponding author.

## References

[1] Mackenzie IR, Neumann M, Bigio EH (2010). Nomenclature and nosology for neuropathologic subtypes of frontotemporal lobar degeneration: an update. Acta Neuropathol.

[2] Kertesz A, Davidson W, Fox H (1997). Frontal behavioral inventory: diagnostic criteria for frontal lobe dementia. Can J Neurol Sci.

[3] Cheran G, Silverman H, Manoochehri M (2018). Psychiatric symptoms in preclinical behavioural-variant frontotemporal dementia in MAPT mutation carriers. J Neurol Neurosurg Psychiatry.

[4] Ismail Z, Smith EE, Geda Y (2016). Neuropsychiatric symptoms as early manifestations of emergent dementia: provisional diagnostic criteria for mild behavioral impairment. Alzheimers Dement.

[5] Ismail Z, Agüera-Ortiz L, Brodaty H (2017). The Mild Behavioral Impairment Checklist (MBI-C): a rating scale for neuropsychiatric symptoms in pre-dementia populations. J Alzheimers Dis.

[6] Mallo SC, Ismail Z, Pereiro AX (2018). Assessing mild behavioral impairment with the mild behavioral impairment-checklist in people with mild cognitive impairment. J Alzheimers Dis.

[7] Mallo SC, Ismail Z, Pereiro AX (2019). Assessing mild behavioral impairment with the mild behavioral impairment checklist in people with subjective cognitive decline. Int Psychogeriatr.

[8] Creese B, Griffiths A, Brooker H (2020). Profile of mild behavioral impairment and factor structure of the Mild Behavioral Impairment Checklist in cognitively normal older adults. Int Psychogeriatr.

[9] Mortby ME, Lyketsos CG, Geda YE (2018). Special issue on mild behavioral impairment and non-cognitive prodromes to dementia. Int Psychogeriatr.

[10] Aguera-Ortiz LF, Lopez-Alvarez J, Del Nido-Varo L (2017). Mild behavioural impairment as an antecedent of dementia: presentation of the diagnostic criteria and the Spanish version of the MBI-C scale for its evaluation. Rev Neurol.

[11] Elefante C, Lattanzi L, Ismail Z (2019). Mild behavioral impairment: presentation of the diagnostic criteria and the Italian version of the MBI-Checklist. Riv Psichiatr.

[12] Cui Y, Dai S, Miao Z (2019). Reliability and validity of the Chinese version of the mild behavioral impairment checklist for screening for Alzheimer's disease. J Alzheimers Dis.

[13] Cieslak A, Smith EE, Lysack J (2018). Case series of mild behavioral impairment: toward an understanding of the early stages of neurodegenerative diseases affecting behavior and cognition. Int Psychogeriatr.

[14] Rascovsky K, Hodges JR, Knopman D (2011). Sensitivity of revised diagnostic criteria for the behavioural variant of frontotemporal dementia. Brain.

[15] Hughes CP, Berg L, Danziger WL (1982). A new clinical scale for the staging of dementia. Br J Psychiatry.

[16] Folstein MF, Folstein SE, McHugh PR (1975). "Mini-mental state". A practical method for grading the cognitive state of patients for the clinician. J Psychiatr Res.

[17] Nasreddine ZS, Phillips NA, Bédirian V (2005). The Montreal Cognitive Assessment, MoCA: a brief screening tool for mild cognitive impairment. J Am Geriatr Soc.

[18] Ricci M, Graef S, Blundo C (2012). Using the Rey Auditory Verbal Learning Test (RAVLT) to differentiate Alzheimer's dementia and behavioural variant fronto-temporal dementia. Clin Neuropsychol.

[19] Cheung RW, Cheung MC, Chan AS (2004). Confrontation naming in Chinese patients with left, right or bilateral brain damage. J Int Neuropsychol Soc.

[20] Greenlief CL, Margolis RB, Erker GJ (1985). Application of the Trail Making Test in differentiating neuropsychological impairment of elderly persons. Percept Mot Skills.

[21] Lawton MP, Brody EM (1969). Assessment of older people: self-maintaining and instrumental activities of daily living. Gerontologist.

[22] Kaufer DI, Cummings JL, Ketchel P (2000). Validation of the NPI-Q, a brief clinical form of the Neuropsychiatric Inventory. J Neuropsychiatry Clin Neurosci.

[23] Hu S, Patten S, Charlton A (2023). Validating the Mild Behavioral Impairment Checklist in a cognitive clinic: comparisons with the Neuropsychiatric Inventory Questionnaire. J Geriatr Psychiatry Neurol.

[24] Taragano FE, Allegri RF, Krupitzki H (2009). Mild behavioral impairment and risk of dementia: a prospective cohort study of 358 patients. J Clin Psychiatry.

[25] Konstantinopoulou E, Aretouli E, Ioannidis P (2013). Behavioral disturbances differentiate frontotemporal lobar degeneration subtypes and Alzheimer's disease: evidence from the Frontal Behavioral Inventory. Int J Geriatr Psychiatry.

[26] Pąchalska M, Bidzan L, Łukowicz M (2011). Differential diagnosis of behavioral variant of fronto-temporal dementia (bvFTD). Med Sci Monit.

[27] Cosseddu M, Benussi A, Gazzina S (2020). Progression of behavioural disturbances in frontotemporal dementia: a longitudinal observational study. Eur J Neurol.

[28] Krudop WA, Kerssens CJ, Dols A (2015). Identifying bvFTD within the wide spectrum of late onset frontal lobe syndrome: a clinical approach. Am J Geriatr Psychiatry.

[29] Cummings JL, Mega M, Gray K (1994). The Neuropsychiatric Inventory: comprehensive assessment of psychopathology in dementia. Neurology.

[30] Godin J, Theou O (2020). The Mild Behavioral Impairment Checklist: a promising tool for assessing mild behavioral impairment in community samples. Int Psychogeriatr.

