# Supplementary material for: Application of the mild behavioral impairment checklist in Chinese patients with the behavioral variant of frontotemporal dementia
Source: Neurol Sci. 2023 Sep 5;45(2):557–64. doi: 10.1007/s10072-023-07049-4 (PMC10791978; doi:10.1007/s10072-023-07049-4)
Supplement: Supplementary file 1 — Supplementary file1 (DOCX 111 KB) [file 10072_2023_7049_MOESM1_ESM.docx]

**Application of the mild behavioral impairment checklist in Chinese patients with the behavioral variant of frontotemporal dementia**

Yue Cui^1^, Li Liu^1^, Min Chu^1^, Kexin Xie^1^, Zhongyun Chen^1^, Haitian Nan^1^, Yu Kong^1^, Tianxinyu Xia^1^, Yingtao Wang^1^, Yihao Wang^1^, Qianqian He^1^, Liyong Wu^1^

^1^Department of Neurology, Xuanwu Hospital, Capital Medical University, Beijing, China

Correspondence to:

Liyong Wu

Department of Neurology

Xuanwu Hospital, Capital Medical University

Changchun Street 45, Beijing 100053, China

Telephone: +86-10-83923051

Fax: None

E-mail: wmywly@hotmail.com

Submited in Neurological Sciences


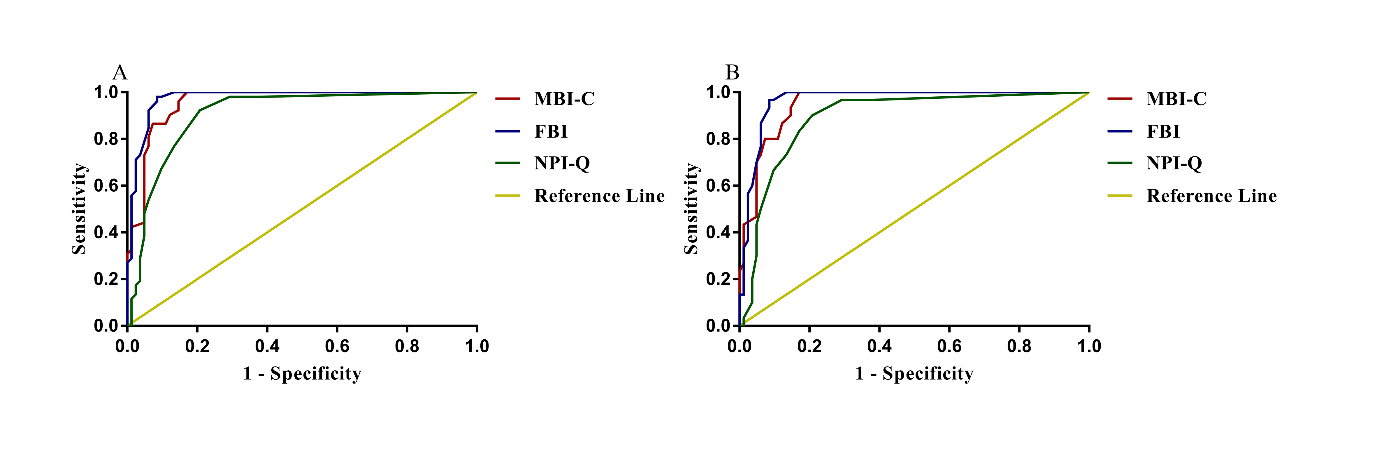
**Supplementary Fig1.** Receiver operating characteristic curves using the MBI-C, NPI-Q, and FBI. (A) differentiating moderate-severe bvFTD from community elderly; (B) differentiating mild bvFTD from moderate-severe bvFTD.

Supplementary Table 1. Optimal Cutoff Points and Validity of the MBI-C, NPI-Q, and FBI.

|  | **Moderate-severe bvFTD**  **and community elderly** | | | |
| --- | --- | --- | --- | --- |
|  | AUC | Cutoff points | Sensitivity | Specificity |
| MBI-C | 0.97 | 12 | 95% | 93% |
| NPI-Q | 0.93 | 2.5 | 95% | 79% |
| FBI | 0.99 | 10.5 | 100% | 93% |

AUC, area under the curve; bvFTD, behavioral variant frontotemporal dementia; FBI, Frontal Behavioral Inventory; MBI, mild behavioral impairment; MBI-C, Mild Behavioral Impairment Checklist; NPI-Q, Neuropsychiatric Inventory Questionnaire
